# Two New Phragmalin-Type Limonoids from *Chukrasia* *tabularis* var. *velutina*

**DOI:** 10.3390/molecules18010373

**Published:** 2012-12-27

**Authors:** Yi Li, Jun Luo, Hui Li, Ling-Yi Kong

**Affiliations:** 1 Testing & Analysis Center, Nanjing Normal University, Nanjing 210023, China; 2 Department of Natural Medicinal Chemistry, China Pharmaceutical University, Nanjing 210009, China

**Keywords:** phragmalin-type limonoids, 16-norphragmalin, carbonate moiety, *Chukrasia tabularis* var. *velutina*

## Abstract

Two new phragmalin-type limonoids with different structural skeletons, chuktabrin K (**1**) and tabulalin J (**2**), were isolated from the stem barks of *Chukrasia tabularis* var. *velutina* in the course of our ongoing research work in this area. Compound **1** was a 16-norphragmalin with an enolic alkyl appendage at C-15, and the carbonate moiety in **1** was also rare in natural organic molecules. The basic skeleton of compound **2** was a D-ring-opened phragmalin. Their structures were elucidated on HR-ESI-MS, ^1^H and ^13^C-NMR, HSQC, HMBC, and ROESY experiments.

## 1. Introduction

**Figure 1:**
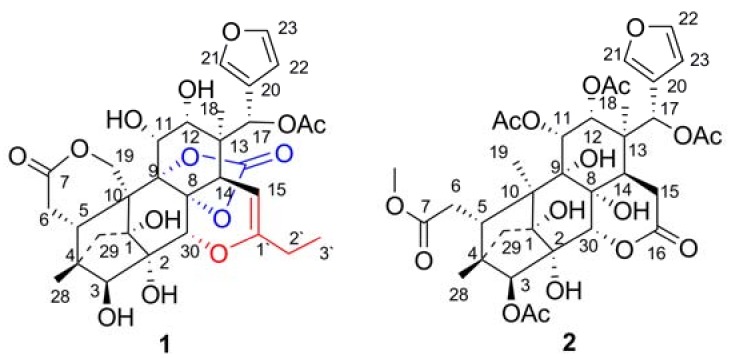
Structures of compounds **1** and **2**.

The stem barks of plants of the genus *Chukrasia*, traditionally used in Southern China to treat cold and fever [1], have been a research focus for natural products chemistry in recent years, and a series of phragmalin-type limonoids with novel and diverse structures have been isolated [2,3,4,5,6]. In our previous research on limonoids from the stem barks of the title plant, many kinds of phragmalins with different skeletons were isolated, such as 16-norphragmalin with ketonic, enolic, or ketal alkyl appendages at C-15 [5,7,8,9], phragmalin orthoesters with enolic alkyl appendages at C-15 [10], phragmalin with an unprecedented 8-oxatricyclo [4,3,11,6] decane moiety [11], and normal phragmalin and its orthoester derivative [12,13,14,15,16]. Further investigation on the phragmalin-type limonoids of this plant led to the isolation of two new phragmalin-type limonoids (Figure 1) with different structural skeletons. Chuktabrin K (**1**) was a rare 16-norphragmalin with enolic alkyl appendage at C-15, and the carbonate moiety in **1** is also rare in natural organic molecules. Tabulalin J (**2**) was a normal phragmalin with a C-16/C-30 δ-lactone ring. Their structures were elucidated on their extensive 1D and 2D spectroscopic analysis (HSQC, HMBC, and ROESY) and HR-ESI-MS. Herein, their isolation and structural elucidation are reported.

## 2. Results and Discussion

**Figure 2:**
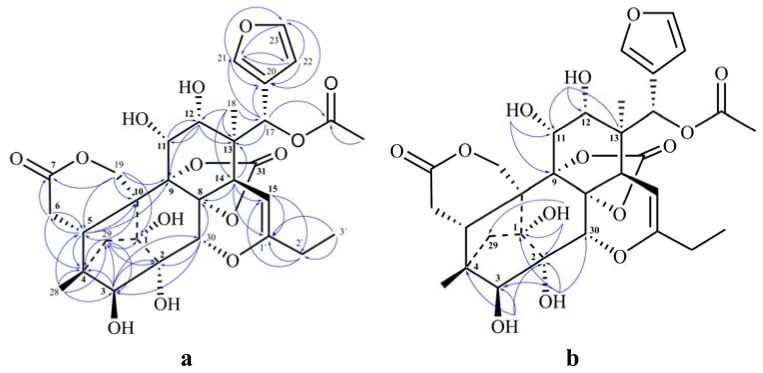
HMBC correlations of compound **1**. (**a**) carbon skeleton (**b**) hydroxyl groups.

Chuktabrin K (**1**) was isolated as a white amorphous powder, and its molecular formula was determined as C_31_H_36_O_14_ by its HRESIMS ion at *m*/*z* 667.1809 ([M+Cl]^−^, C_31_H_36_O_14_Cl; calc. 667.1799). Characteristic 1D-NMR spectra, *i.e.*, three upfield proton signals at δ_H_ 6.42, 7.59, and 7.68 and a set of double proton signals at δ_H_ 1.92 and 1.46 with an 11.0 Hz coupling constant in the ^1^H-NMR (Table 1), and four olefinic carbons at δ_C_ 109.9, 121.5, 141.1, and 143.7 in the ^13^C-NMR (Table 1), indicated that compound **1** was a phragmalin-type limonoid possessing an α,β-substituted furan ring and a 4,29,1-bridge moiety [8]. The presence of a carbon signal at δ_C_ 92.4 showing a HSQC correlation with the proton signal at δ_H_ 4.67 and two down-field carbon signals at δ_C_ 152.0 and 152.8 suggested that compound **1** was a 16-norphragmalin with an enolic alkyl appendage at C-15 and a characteristic carbonate moiety like chuktabrins C-H [8]. The obvious HMBC correlations (Figure 2a) from a set of ethyl proton signals [δ_H_2.10, q, (7.5), 2H; δ_H_ 1.04, t (7.5), 3H] and proton signal of H-15 (δ_H_ 4.67) to a carbon signal at δ_C_ 152.8 (C-1') indicated that a propionyl group was attached at C-15 biosynthetically as in the chuktabrins C-H [8].

Comparison of the NMR data between **1** and chuktabrin H indicated that the former was a deacetyl derivative of the latter, which was also confirmed from the molecular formula by the absence of one C_2_H_2_O unit. An obvious HMBC correlation (Figure 2a) from H-17 (δ_H_ 5.79) to the ^13^C signal for an acetoxyl group (MeCOOR) at δ_C_ 167.9 suggested that the only acetyl group was located at OH-17. Hitherto, the planar structure of compound **1** was determined, except for the location of the carbonate moiety and the ether linkage of C-1' due to a lack of direct HMBC evidence. Hydroxyl groups must be connected at C-1, C-2, C-3, C-11 and C-12 due to the observed correlations between the 1-OH signal (δ_H_ 5.35) to the ^13^C signal for C-1 at δ_C_ 82.3 and C-2 at δ_C_ 74.0; 2-OH (δ_H_ 4.23) to C-1 at δ_C_ 82.3, C-3 at δ_C_ 85.7 and C-30 at δ_C_ 65.7; 3-OH (δ_H_ 5.92) to C-2 at δ_C_ 74.0, C-3 at δ_C_ 85.7 and C-4 at δ_C_ 43.1; 11-OH (δ_H_ 5.67) to C-11 at δ_C_ 67.3 and C-9 at δ_C_ 86.6; 12-OH (δ_H_ 5.14) to C-11 at δ_C_ 67.3, C-12 at δ_C_ 73.4 and C-13 at δ_C_ 43.9. The ^1^H signal for H-30 at δ_H_ 4.57 (δ_C_ 65.7) showed correlation to the ^13^C signal at δ_C_ 83.2 (C-8). Thus, these correlation required the presence of -OR substituent at C-8, C-9 and C-30. The ^13^C-NMR data for C-30, C-9 and C-8 were similar to those for chuktabrin C, which was determined by single-crystal X-ray diffraction [8], determining the position of enol ether at C-30 and the carbonate moiety at C-8 and C-9.

**Figure 3:**
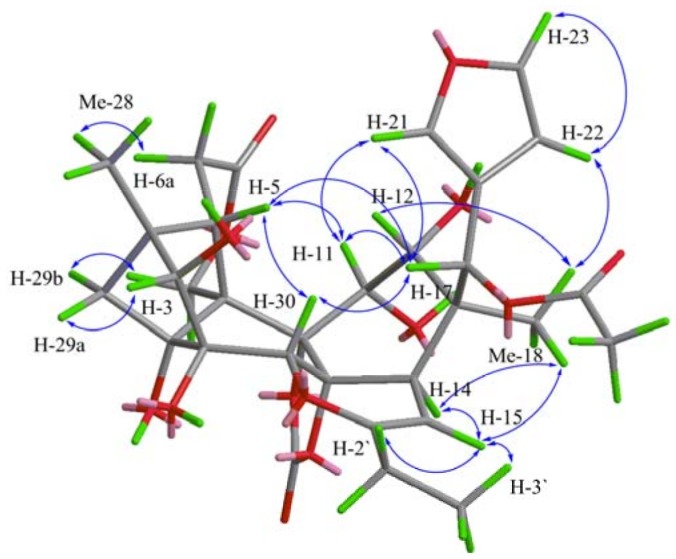
Key ROESY correlations of compound **1**.

The key ROESY correlations (Figure 3), from H-5 to H-11, H-17, and H-21, H-17 to H-5, H-11, H-21, and H-30, Me-18 with H-14 and H-22, H-29a with H-3, and H-29b with H-19b, indicated that the relative stereochemistry of the key asymmetric carbons of **1** was well matched with those of chuktabrin C obtained by X-ray crystallography [8]. Thus, the structure of **1** was established as shown in Figure 1, namely as a 12-deacetyl derivate of chuktabrin H [8].

**Table 1:** ^1^H (500 MHz) and ^13^C (125 MHz) NMR data of **1** and **2** in DMSO-*d*6.

No.	1		2	
	δ_H_ (multi, *J* in Hz)	δ_C_	δ_H_ (multi, *J* in Hz)	δ_C_
1		82.3		82.9
2		74.0		74.8
3	3.35 (d 5.5)	85.7	4.73 (s)	86.1
4		43.1		43.9
5	2.44 (m )	36.7	2.60 *	39.2
6a	2.38 (dd 16.0, 3.3)	29.6	1.94 * 2.47(dd 18.0, 11.0)	31.9
6b	2.94 (dd 16.0, 6.0)			
7		172.8		172.4
8		83.2		71.7
9		86.6		76.4
10		48.1		51.8
11	3.88 (dd 3.0, 8.6)	67.3	5.11 (d 3.0)	71.2
12	3.17 (dd 3.0, 5.0)	73.4	4.96 (d 3.0)	70.8
13		43.9		42.3
14	2.52 (d 4.5 )	43.4	2.60 *	40.3
15a	4.67 (d 4.5)	92.4	2.76 (d 18.5)	27.8
15b			2.88(dd 18.5, 9.0)	
16				168.9
17	5.79 (s)	67.5	6.00(s)	70.2
18	1.29 (s, 3H)	17.1	1.04 (s 3H)	18.4
19a	4.57 (d 12.5)	67.9	1.19 (s 2H)	15.3
19b	5.27(d 12.5)			
20		121.5		121.4
21	7.59 (s)	141.1	7.75 (s)	141.7
22	6.42 (s)	109.9	6.57 (t 1.0)	109.7
23	7.68 (s)	143.7	7.65 (t 1.5)	143.2
28	0.88 (s 3H)	14.9	0.70 (s 3H)	14.6
29a	1.46 (d 11.0)	41.4	1.51 (d 11.0)	40.8
29b	1.92 (d 11.0)		1.98(d 11.0)	
30	4.57 (s)	65.7	4.98 (s)	74.4
31		152.0		
1'		152.8		
2'	2.10 (q 7.5)	26.0		
3'	1.04 (t, 7.5)	10.9		
7-OMe			3.62 (s 3H)	51.4
1-OH	5.35 (s)		6.46 (s)	
2-OH	4.23 (s)		5.06 (s)	
3-OH	5.92 (d5.5)			
8-OH			6.64 (s)	
9-OH			4.42 (s)	
11-OH	5.67 (d 8.6)			
12-OH	5.14 (d 5.0)			
3-OAc				170.0
			2.22 (s 3H)	20.4
11-OAc				170.1
			2.03 (s 3H)	20.5
12-OAc				168.9
			1.91 (s 3H)	20.4
17-OAc		167.9		168.6
	1.91 (s 3H)	20.7	1.97 (s 3H)	20.8

* Resonance pattern unclear due to overlapping.

**Figure 4:**
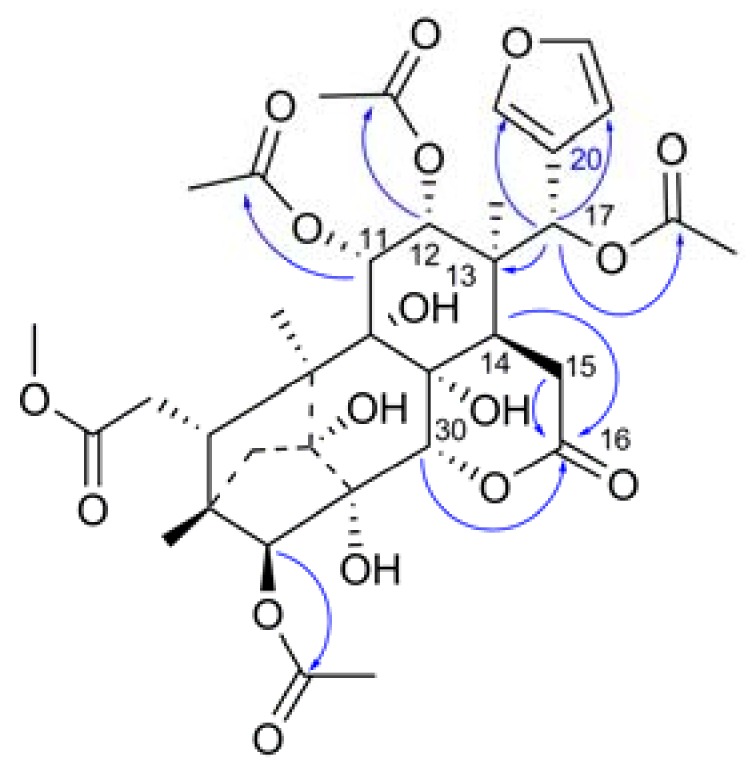
Key HMBC correlations of compound **2**.

Tabulalin J (**2**), was obtained as a white amorphous powder, and its molecular formula was established as C_35_H_44_O_17_ by the HRESIMS ion at *m*/*z* 735.2516 ([M−H]^−^, C_35_H_43_O_17_; calc. 735.2506). The similarity of the ^1^H and ^13^C-NMR spectroscopic data of **2** (Table 1) to those of tabulalin A, a phragmalin-type limonoid isolated in our previous research [13], indicated that these two molecules possessed the same carbon framework. Obvious HMBC correlations (Figure 4a) from H-17 (δ_H_ 6.00) to the ^13^C signal for the acetoxyl group (MeCOOR) at δ_C_ 168.6 and H-30 (δ_H_ 4.98) to C-16 (δ_C_ 168.9) indicated that compound **2** possesses the same phragmalin skeleton with a C-16/C-30 δ-lactone ring as tabulalin A [13]. Comparison of the NMR data and molecular formula suggested that **2** was a bisacetyl derivative of tabulalin A [13]. A significant downfield shift for H-11 (δ_H_ 5.11) and H-12 (δ_H_ 4.96), when compared with tabulalin A, determined the position of the acetoxyl groups at C-11 and C-12. In comparison with the ^13^C-NMR spectrum of the parent compound tabulalin A (C-9 δ_C_ 79.3, C-11 δ_C_ 67.7, C-12 δ_C_ 76.0), the acetylation of hydroxyl groups at C-11 and C-12 resulted in 2.9 ppm upfield shifts for the C-9 and 5.2 ppm for C-12 resonances, and 3.5 ppm downfield ships for C-11 position, confirming the acetoxyl groups at C-11 and C-12. Thus, the planar structure of **2** was determined. The relative configuration of **2** was determined to be the same as tabulalide A [13] by its key ROESY correlations, such as from H-11 to H-5, H-12, H-17, and H-30, from H-17 to H-21 and H-30, from H-21 to H-12 and H-30, from H-30 to H-5, from H-3 to Me-28 and H-29a, H-29b to Me-19. Thus, the structure of **2** was demonstrated as 11,12-bisacetyl derivative of tabulalin A [13].

## 3. Experimental

### 3.1. General

Optical rotations were measured with a JASCO P-1020 polarimeter. IR (KBr disks) spectra were recorded on a Bruker Tensor 27 spectrometer. NMR spectra were recorded on Bruker ACF-500 NMR instrument, (^1^H: 500 MHz, ^13^C: 125 MHz), with TMS as internal standard. Mass spectra were obtained on a MS Agilent 1100 Series LC/MSD ion-trap mass spectrometer (ESIMS) and a Agilent UPLC (1290)-TOFMS (6520B) (HR-ESI-MS), respectively. All solvents used in column chromatography were analytical grade (Jiangsu Hanbang Science and Technology Co., Ltd., Huanan, China). Silica gel (Qingdao Haiyang Chemical Co., Ltd., Qingdao, China), Sephadex LH-20 (Pharmacia, Uppsala, Sweden), and RP-C_18_ (40–63 μm, FuJi, Aichi, Japan) were used for column chromatography. Preparative HPLC was carried out using an Agilent 1100 Series instrument (Agilent Technologies, Santa Clara, CA, USA) equipped with a Shim-park RP-C_18_ column (20 × 200 mm) and a 1100 Series multiple wavelength detector.

### 3.2. Plant Material

The air-dried stem bark of *Chukrasia tabularis* var. *velutina* (Wall.) King was collected from Xishuangbanna, Yunnan Province, People’s Republic of China, in March 2007, and was authenticated by Professor Mian Zhang of the Research Department of Pharmacognosy, China Pharmaceutical University. A voucher specimen (No. 2006-MML) has been deposited in the Department of Natural Medicinal Chemistry, China Pharmaceutical University.

### 3.3. Extraction and Isolation

The air-dried stem bark (10 kg) was extracted by refluxing with 95% ethanol (40 L) three times. The EtOH extract was concentrated under reduced pressure (2,000 g) and then extracted with CHCl_3_ to give a chloroform extract (300 g). The oily chloroform extract was dissolved in 2 L MeOH/H_2_O (50:50, v/v) and then extracted with petroleum ether (6 L, 60–90 °C, ×3). After removal of the fatty components, 210 g of extract were obtained, which was subjected to silica gel column chromatography eluting with CHCl_3_/MeOH in a gradient from 1:0 to 1:2 to afford eight fractions (Fractions A–H). Fraction E (20 g) was chromatographed on a column of reversed-phase C_18_ silica gel eluted with MeOH/H_2_O (4:6 to 7:3) to give six sub-fractions (Fractions E1–6). Fraction E6 (7 g) was chromatographed on a column of reversed-phase C_18_ silica gel eluted with MeOH/H_2_O (2:3 to 7:3) to give four sub-fractions (Fractions E6a–d), Fraction E6c was separated by prep-HPLC using MeOH/H_2_O (55:45) as the mobile phase to give compound **2** (3 mg). Fraction F (13 g) was chromatographed on a column of silica gel eluted successively with a gradient of petroleum ether/EtOAc (1:1 to 1:4) to give four sub-fractions (Fractions F1–4). Fraction F3 was chromatographed on a column of reversed-phase C_18_ silica gel eluted with MeOH/H_2_O (2:3 to 7:3) to give four sub-fractions (Fractions F3a–d). Fraction F2a was separated by preparative HPLC using CH_3_OH/H_2_O (52:48, 10 mL/min) as the mobile phase to give **1** (4 mg).

*Chuktabrin K* (**1**), White, amorphous powder; [${\left[\alpha \right]}_{D}^{25}$ +46 (*c* 0.10, CH_3_OH); IR (KBr) cm^−1^: 3450, 2976, 1800, 1736, 1640, 1378, 1238, 1033; ^1^H and ^13^C-NMR, see Table 1; negative ESIMS *m/z*: 667.3 [M+Cl]^−^ (100); positive ESIMS *m/z*: 650.2 [M+NH_4_]^+^ (100); HRESIMS *m/z*: 667.1809 ([M+Cl]^−^, C_31_H_36_O_14_Cl; calc. 667.1799).

*Tabulalin J* (**2**), White, amorphous powder; ${\left[\alpha \right]}_{D}^{25}$ −23 (*c* 0.15, CH_3_OH); IR (KBr) cm^−1^: 3450, 2973, 1740, 1640, 1463, 1376, 1242, 1169; ^1^H and ^13^C-NMR, see Table 1; negative ESIMS *m/z*: 771.1 [M+Cl]^−^ (100); positive ESIMS *m/z*: 754.3 [M+NH_4_]^+^ (100); HRESIMS *m/z*: 735.2516 [M−H]^−^ (calcd: C_37_H_45_O_17_, 735.2506).

## 4. Conclusions

Two new phragmalin-type limonoids with different structure skeletons, chuktabrin K (**1**) with a 16-norphragmalin skeleton and tabulalin J (**2**) with a normal phragmalin skeleton, were isolated from the stem barks of *Chukrasia tabularis* var. *velutina*. The complex and new structures of these two phragmalins represent new additions to the molecular diversity of natural organic limonoid molecules.
